# Supplementary figures and images for: CureSCi Metadata Catalog—Finding and harmonizing studies for secondary analysis of hydroxyurea discontinuation in sickle cell disease
Source: PLoS One. 2025 Apr 23;20(4):e0309572. doi: 10.1371/journal.pone.0309572 (PMC12017531; doi:10.1371/journal.pone.0309572)

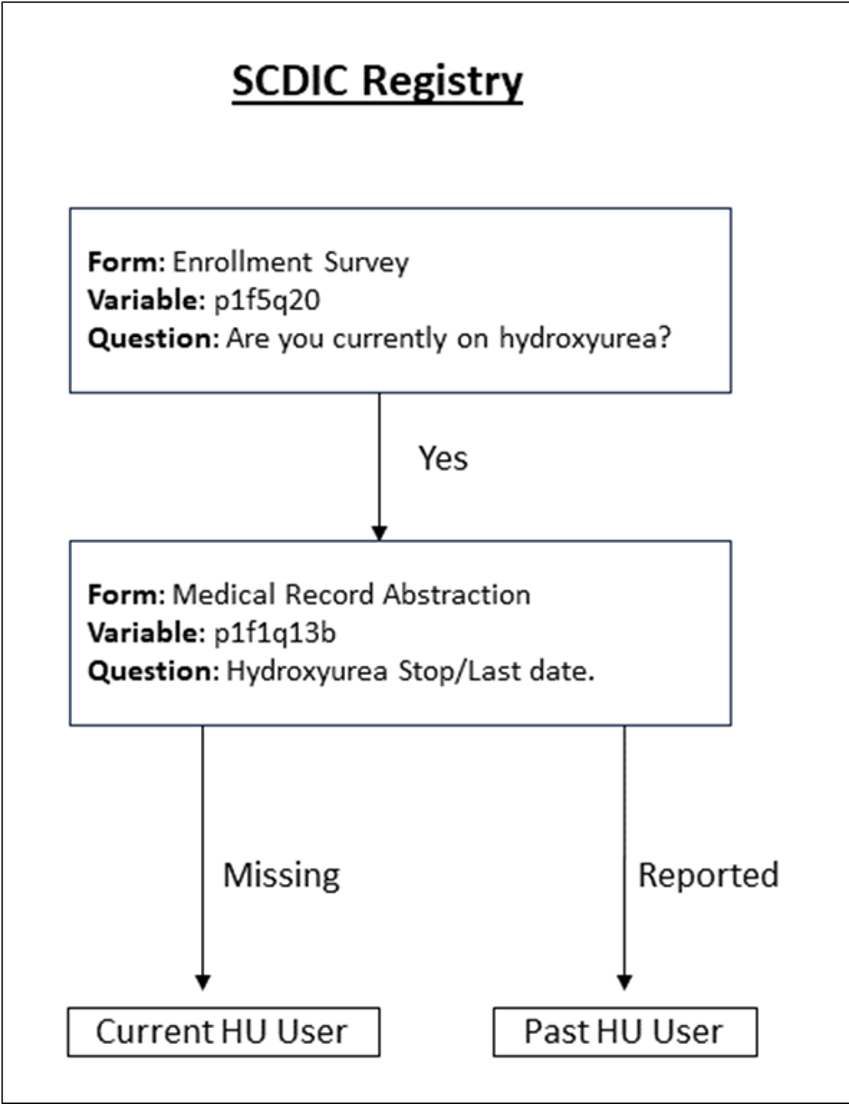

Supplement: S1 Fig — (PNG) [file pone.0309572.s005.png]
